# Exercise mediated protection of diabetic heart through modulation of microRNA mediated molecular pathways

**DOI:** 10.1186/s12933-016-0484-4

**Published:** 2017-01-13

**Authors:** Jason Kar Sheng Lew, James T. Pearson, Daryl O. Schwenke, Rajesh Katare

**Affiliations:** 1 Department of Physiology, HeartOtago, University of Otago, 270, Great King Street, Dunedin, 9010 New Zealand; 2 Department of Cardiac Physiology, National Cerebral and Cardiovascular Center Research Institute, Suita, Osaka Japan; 3 Biomedicine Discovery Institute and Department of Physiology, Monash University, Clayton, Australia

**Keywords:** Exercise, Diabetic heart disease, Hyperglycaemia, Insulin resistance, MicroRNA, Cardioprotection, Cross-talk effect

## Abstract

Hyperglycaemia, hypertension, dyslipidemia and insulin resistance collectively impact on the myocardium of people with diabetes, triggering molecular, structural and myocardial abnormalities. These have been suggested to aggravate oxidative stress, systemic inflammation, myocardial lipotoxicity and impaired myocardial substrate utilization. As a consequence, this leads to the development of a spectrum of cardiovascular diseases, which may include but not limited to coronary endothelial dysfunction, and left ventricular remodelling and dysfunction. Diabetic heart disease (DHD) is the term used to describe the presence of heart disease specifically in diabetic patients. Despite significant advances in medical research and long clinical history of anti-diabetic medications, the risk of heart failure in people with diabetes never declines. Interestingly, sustainable and long-term exercise regimen has emerged as an effective synergistic therapy to combat the cardiovascular complications in people with diabetes, although the precise molecular mechanism(s) underlying this protection remain unclear. This review provides an overview of the underlying mechanisms of hyperglycaemia- and insulin resistance-mediated DHD with a detailed discussion on the role of different intensities of exercise in mitigating these molecular alterations in diabetic heart. In particular, we provide the possible role of exercise on microRNAs, the key molecular regulators of several pathophysiological processes.

## Background

Type-2 diabetes mellitus (T2DM) has emerged as one of the most serious health problems in modernized society, affecting 387 million people world wide [1]. Of concern, over 68% of diabetic patients will develop some form of heart disease or stroke that will ultimately prove fatal [2].

A link between DM and cardiovascular disease is undisputable. Indeed, hyperglycaemia, hypertension, dyslipidemia and insulin resistance collectively impact on the myocardium of diabetic patients, triggering several early pathophysiological molecular, structural and myocardial abnormalities [3–9], which may include but are not limited to coronary endothelial and vascular dysfunction, and left ventricular remodelling and dysfunction. Due to these underlying dysfunctions, DM increases the risk for the development of a spectrum of cardiovascular disease in people with DM as compared to their non-DM counterparts. The highly cited Framingham Heart Study (FHS) showed that diabetes independently increased the risk of coronary heart disease (CHD) in men by 66% and in women by 203% when followed up for 20 years, after adjusting for the effects of age, smoking, cholesterol and blood pressure, respectively [10]. Based on the findings of FHS, it was suggested that the duration of diabetes significantly increased the risk of developing CHD and mortality [11]. More recently, a prospective study covering a 55 year span showed that, while mortality has significantly declined over time in both men and women with DM, these mortality rates still remained almost twofold higher compared to those without DM [12].

This review provides an overview (a) of the underlying mechanisms of hyperglycaemia- and insulin resistance-mediated diabetic heart disease (DHD), juxtaposing these key factors in the pathological setting with current knowledge of exercise-induced cardioprotection, and (b) of how exercise can prevent DHD through potential miR cross-talk effects, and finally (c) on the potential roles of miRs as biomarkers to demonstrate the benefit of exercise.

## Diabetic heart disease

In 1980, the term diabetic heart disease emerged as a variable combination of coronary atheroma, cardiomyopathy, microangiopathy and autonomic neuropathy [13]. It was proposed that heart disease in diabetes is not synonymous with coronary artery disease because the increased incidence of coronary risk factors in diabetes has failed to account for the observed cardiovascular mortality [13]. In agreement, the National Institute of Health (NIH) also defined DHD as the presence of heart disease specifically in diabetic patients that encompasses coronary heart disease, heart failure and/or cardiomyopathy [14]. Of note, DHD is a broad definition encapsulating a diverse range of myocardial diseases in the diabetic population, due to the fact that (a) the aetiology is varying among individuals (e.g.: genetic susceptibility, environmental factor etc.) and (b) the mechanisms of DHD are poorly understood and defined. Hence, DHD can be a distinct clinical entity and should not be limited to a particular type of myocardial disease, rather, characterized as a myocardial disease in people with T2DM that cannot be ascribed to the individual effects of coronary artery disease, hypertension or other known cardiac disease [15].

## Pathogenesis of DHD

**Fig. 1:**
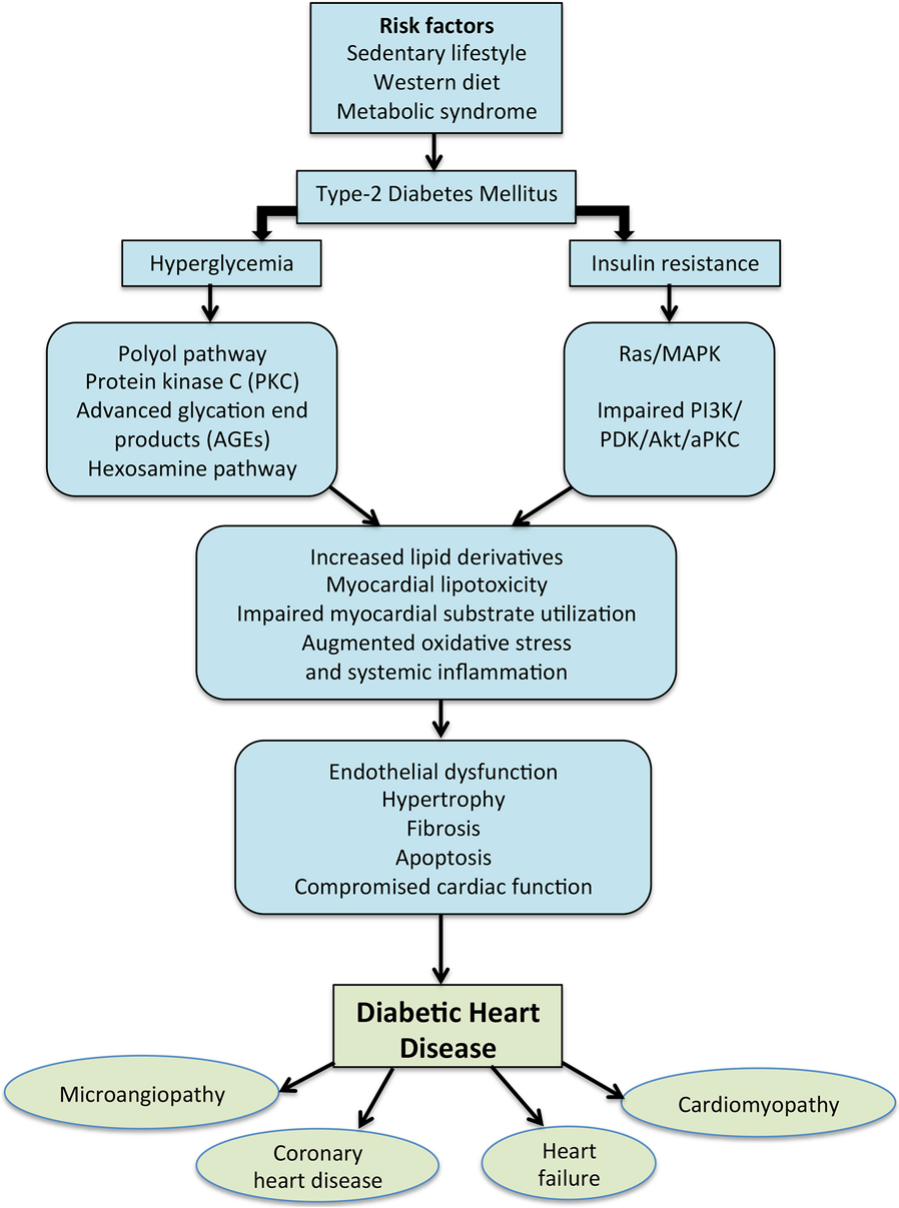
Pathogenesis of diabetic heart disease

The aetiology of DHD is multifactorial and remains unresolved. However, increasing evidence suggests that hyperglycaemia and insulin resistance are linked to the development of DHD [4, 5, 16–24]. Additional risk factors such as hypertension, obesity, hypercholesterolemia, coronary artery disease, microvascular disease and cardiac neuropathy are also known to contribute to the progression of DHD. Although a complete molecular description of DHD is beyond the scope of this review (Detailed review in [25]), a basic understanding on hyperglycaemia- and insulin resistance-mediated pathological events in the development of DHD is important in order to appreciate exercise-mediated protection of DHD as shown in Fig. 1.

### Hyperglycaemia-induced cardiovascular dysfunction

Hyperglycaemia induces activation of polyol pathway (through the activation of aldose reductase), protein kinase-C pathway (PKC), advanced glycation end products (AGEs) pathway and hexosamine pathway, all of which have the potential to increase myocardial oxidative stress [26, 27] and cardiovascular dysfunction in diabetes.

Increased intracellular glucose concentration increases aldose reductase activity, which uses excess nicotinamide adenine dinucleotide phosphate (NADPH) as a cofactor to convert glucose to sorbitol, resulting in the depletion of intracellular NADPH [28]. This eventually reduces the generation of reduced glutathione (GSH), an intracellular antioxidant [29, 30]. As a result, the net production of GSH decreases, hindering the antioxidant capacity to counteract the augmented intracellular oxidative stress caused by high glucose. Altered aldose reductase activity has been reported to predispose the myocardium to ischemic insult [31]. Indeed, inhibition of aldose reductase was able to protect isolated type-1 diabetic rat hearts from ischemia reperfusion injury by preserving high-energy phosphates and maintaining a lower cytosolic NADH/NAD + ratio [31]. In a clinical study, one-year of aldose reductase inhibition treatment was able to stabilize and partially reverse left ventricular abnormalities in diabetics with neuropathy [32].

The hyperglycaemia-mediated increase in total diacylglycerol (DAG) from the glycolytic intermediate, glycerol-3-phosphate (G3P), can trigger the activation of DAG-protein kinase C (DAG-PKC) [33]. Intracellular hyperglycaemia activates PKC-β and -δ isoforms, which induce activation of pro-inflammatory genes [e.g. p38 and nuclear factor kappa-light-chain-enhancer of activated B cell (NK-κB)] and microvascular matrix remodelling, impair vascular permeability and inhibit endothelial nitric oxide synthase (eNOS) [34–38]. In addition, this pathway also activates NADPH oxidase, causing an intracellular overproduction of reactive oxygen species (ROS) [39, 40]. In support to this notion, transgenic mice with cardiac-specific overexpression of PKC-β_2_ exhibited cardiac hypertrophy, fibrosis, dystrophic calcification and increased cardiomyocytes death [41].

The increased concentration of glycolytic intermediates such as G3P, glucose-6-phosphate and fructose accelerate the production of advanced glycation end products (AGEs) through a non-enzymatic reaction between proteins, lipids or nucleic acids (reviewed in [42]). Increased AGEs have been demonstrated to contribute to the pathogenesis of DHD by altering the functional and elastic properties of the blood vessels, vascular tone and extracellular matrix [43–46]. In fact, binding of AGE to its receptor (RAGE) on endothelial cells, smooth muscle cells, and macrophages triggers a series of molecular pathways, contributing to the activation of inflammatory signalling cascades, oxidative stress, increased vascular permeability, atherogenesis and vasoconstriction, leading to diverse vascular dysfunction [47–51]. To support this notion, in a series of elegant studies conducted by Zitman-Gal et al., the treatment of AGE (diabetic-like environment) on endothelial cells (ECs) and vascular smooth muscle cells was able to induce significant expression of various inflammatory markers such as Kruppel-like factor, IL-6, IL-8 and thioredoxin-interacting protein (TXNIP), suggesting a direct role for the adverse effect of AGE in the development of diabetic vascular complications [52–54]. In addition, the level of circulating AGE has been suggested as an independent predictor of the prognosis for heart failure patients [55]. Elevated AGE level has also been suggested to be highly correlated with insulin resistance in T2DM [56].

Glucose metabolism through hexosamine pathway is relatively low (1-3%) in physiological conditions. In this pathway, fructose-6-phosphate is first converted to glucosamine-6-phosphate (GlcN-6-P) by glutamine:fructose-6-phosphate amidotransferase (GFAT). Subsequently, GlcN-6-P is metabolized to form uridine diphosphate (UDP)-*N*-acetylglucosamine. Enzyme O-linked *N*-acetylglucosamine transferase (O-GlcNAc) utilizes UDP-*N*-acetylglucosamine to modify serine and threonine on cytosolic and nuclear proteins (reviewed in [57]). Importantly, elevated glucose concentration is known to induce O-GlcNAc expression [26, 58]. Altered O-GlcNAcylation has been shown to associate with impaired Ca^2+^ handling protein [59], fibrosis [22], insulin signalling [60, 61], cardiomyocyte hypertrophy [62], impaired relaxation and vascular function [63].

### Insulin resistance-mediated cardiovascular dysfunction

Diabetes impairs the PI3K-mediated pro-survival signalling cascade, while preserving the mitogenic Ras/MAPK-dependent pathway [74–76], thereby shifting the balance in favour of atherogenic and mitogenic actions of insulin. Insulin receptor substrate-1 (IRS) is required for the activation of phosphoinositide-3 kinase/phosphoinositide-dependent kinase 1/protein kinase B/atypical protein kinase C (PI3K/PDK1/Akt/aPKC) cascade, which regulates translocation of glucose transporter (GLUT)-1 and -4 proteins [64, 65], nitric oxide production [66], apoptosis [67], autophagy [68] and fat metabolism [69]. In contrast, activation of Ras-mitogen-activated protein kinase-dependent pathway (Ras/MAPK) (reviewed in [70]) promotes cellular differentiation, proliferation, apoptosis through its downstream effectors: c-Jun N-terminal kinase (JNK), extracellular signal-regulated kinase (ERK) and p38 MAPK (reviewed in [71–73]).

#### Effects of insulin resistance on myocardial lipotoxicity

Excessive FFA and lipid oxidation are often seen in diabetic heart, causing them to lose the flexibility to switch its source of energy between FFA and glucose [77, 78]. This reduction in insulin-mediated glucose uptake forces cardiomyocytes to heavily rely on FFA oxidation for their energy source. Since myocardial tissue utilizes more oxygen to metabolize a single molecule of FFA compared to glucose molecule, the oxygen cost to produce adenosine triphosphate (ATP) in FFA oxidation is higher than glucose metabolism [21, 79]. Of importance, increased oxygen cost reduces the cardiac efficiency that is associated with the development of dilated cardiomyopathy, heart failure and ventricular dysfunction [20, 79–81].

#### Effects of insulin resistance on oxidative stress

Insulin resistance is also associated with the overproduction of oxidants due to the proportional increase of electron donors to the mitochondrial electron transport chain during FFA oxidation, which inactivates two important antioxidants: prostacyclin synthase and eNOS [82]. In line with this, inhibition of FFA release from adipocytes and inhibition of the rate-limiting enzyme for FFA oxidation completely reversed ROS production in insulin resistant but not in non-diabetic rodent models [82]. Consequently, overproduction of superoxide inhibits IRS-1-induced PI3K-dependent pathway activation [83], thereby suppressing the pro-survival pathways (reviewed in [84, 85]).

#### Effects of insulin resistance on systemic inflammation

Adipokines are cytokines that are constantly produced by adipocytes, such as tumour necrosis factor- alpha (TNF-α), interleukin-6 (IL-6) and angiotensinogen, In addition to adipocyte sources, FFA-induced and electron uncoupling-evoked ROS can also directly stimulate proinflammatory cytokine production through the activation of nuclear factor kappa B (NFκB) [86]. Eventually, overproduction of these cytokines is considered to inhibit insulin-mediated metabolic effects through several mechanisms. Firstly, TNF-α can attenuate PI3K/Akt-dependent cell survival signalling through phosphorylation of IRS-1 at Serine^307^ [87] and activation of p38 MAPK and I-kappaB kinase β (IKKβ) [88]. Secondly, both TNF-α and IL-6 can stimulate suppressor of cytokine-signalling-1 and -3 proteins (SOCS-1, -3) expression [89, 90]. SOCS protein then inhibit the coupling of IRS-1 and PI3K proteins either by ubiquitination of IRS proteins for proteasomal degradation [91] or through the inhibition of tyrosine phosphorylation of IRS protein [92]. Taken together, inhibition of upstream mediators of PI3K protein induced by augmented cytokines and ROS can result in the suppression of insulin-mediated metabolic regulation.

## Pharmacological Intervention for DHD

The current clinical treatment for diabetes-associated myocardial dysfunction is solely dependent on a ‘cocktail’ of drugs and ‘symptomatic treatment’ approaches [93–95]. For instance, patients with diabetes are often prescribed a plethora of drugs, which include multiple glucose lowering agents, antihypertensive drugs, anti-cholesterol and/or aspirin for cardiac health [93–95]. The use of glucose lowering agents may decrease the risk of microvascular complications such as nephropathy, retinopathy and neuropathy [96, 97]. However, despite their long clinical treatment for diabetes, their efficacies in the improvement of DHD still remain speculative. In essence, there is still no single drug that specifically and effectively treats DHD, primarily due to the fact that the mechanism(s) underpinning DHD are poorly understood and are multi-factorial. Table 1 summarizes the common medications used in combination to alleviate the symptoms of DHD. These medications primarily target DHD symptoms and sometimes act as secondarily to reduce the risk of diabetes complications.

**Table 1:** Summary of common medications used in combination to alleviate the symptoms of DHD

Target	Drug class	Mechanism of action(s)	Primary outcome(s)	Associated outcome(s)	Ref.
Blood glucose	Biguanides (e.g.: metformin)	Activates AMP kinase subunit beta-1	Decreases blood glucose Decreases intestinal absorption of glucose Increases peripheral glucose uptake and utilization Lower hepatic gluconeogenesis Increases insulin sensitivity	Reduces all-cause mortality and CVD events Lower risk of heart failure	[93, 232–234]
	Glucagon-like peptide-1 (GLP-1) (e.g.: Exenatide, Liraglutide, Albiglutide)	Functional analog of the human incretin Glucagon-Like Peptide-1 (GLP-1) Activates GLP-1 receptor	Enhances glucose-dependent insulin secretion Reduces gastric emptying rate Reduces food intake	Reduces post-prandial glucose Reduces body weight Possibly reduces CVD risks	
	Sulfonylureas (e.g.: Glimepiride, Glyburide, Glipizide)	Closure of ATP-sensitive inward rectifier potassium channel-1 and-11 on β-cells Closure of ATP-binding cassette sub-family C member 8 on β-cells	Decreases blood glucose Stimulates insulin secretion Increases peripheral insulin sensitivity	Decreases microvascular risks	
	Thiazolidinediones (e.g.: Pioglitazone, Rosiglitazone)	Activates peroxisome proliferator activated receptors (PPAR) Regulates the transcription of insulin-responsive genes	Decreases blood glucose Increases insulin sensitivity	Increases high-density lipoprotein Decreases triglycerides Possibly reduces CVD risks	
	Insulin (e.g.: Glulisine, Lispro, Aspart, Glargine, Detemir)	Activates insulin receptors (PI3K/Akt/PKC cascade)	Stimulates insulin-dependent glucose transporters Promotes glucose disposal Lower hepatic gluconeogenesis Reduces ketogenesis	Decreases microvascular risks	
Blood pressure	Angiotensin-converting enzyme (ACE) inhibitors (e.g.: Benazepril, Lisinopril, Enalapril, Fosinopril,)	Inhibits angiotensin-converting enzyme Suppresses the conversion of angiotensin-I to angiotensin-II	Reduces plasma angiotensin-I Reduces vasopressor activity Decreases aldosterone secretion Suppresses vasoconstriction Lower blood pressure	Adjunctive therapy for congestive heart failure May be used to delay the progression of renal disease	[93, 234, 235]
	Calcium channel blockers (e.g.: Amlodipine, Lacidipine)	Inhibits influx of calcium ions on L-type calcium channels Decreases arterial smooth muscle contractility	Lower blood pressure Dilates coronary and systemic arteries Promotes coronary blood flow Decreases cardiac output	Adjunctive therapy for coronary syndrome	
	Diuretics (e.g.: Polythiazide, Chlorothiazide, Chlorthalidone, Bumetanide)	Inhibits active chloride reabsorption Increases sodium and water excretion	Decreases preload Lower blood pressure	Adjunctive therapy for edema associated with congestive heart failure, hepatic and renal disease	
	Beta blockers (e.g.: Atenolol, Metoprolol, Carvedilol, Nadolol, Acebutolol)	Inhibits β1-adrenegic receptor	Decreases heart rate Decreases cardiac output Lower blood pressure Suppresses vasoconstriction	Not primary hypertensive therapy Adjunctive therapy for heart failure Prevention for patients with underlying ischemic heart disease	
Blood cholesterol	Statin (e.g.: Atorvastatin, Simvastatin, Pravastatin, Fluvastatin)	Inhibits hepatic enzyme HMG-CoA reductase Inhibits cholesterol biosynthesis Increases hepatic uptake of LDL	Decreases total cholesterol, LDL, triglycerides, apolipoprotein B Increases HDL	Adjunctive therapy for CHD Primary prevention to reduce risk of myocardial infarction, stroke and coronary syndrome	[93, 234, 236]
	Fibrates (e.g.:Gemfibrozil, Fenofibrate)	Increases the activity of extrahepatic lipoprotein lipase (LL) Increases lipoprotein triglyceride lipolysis Activates Peroxisome proliferator-activated receptor-alpha (PPARα)	Increases triglyceride clearance Decreases Chylomicrons, apolipoprotein B		

According to the United Kingdom Prospective Diabetes Study (UKPDS), intensive blood glucose control (with fasting blood glucose <6 mmol/L) in people with T2DM over a period of 10-years significantly reduced microvascular complications, as well as the deaths associated with diabetes-related complications such as hyperglycaemia, angina and heart failure [96]. In contrast, large-scale clinical studies such as Action in Diabetes and Vascular Disease (ADVANCE) [97], Action to Control Cardiovascular Risk in Diabetes (ACCORD) [98] and Veterans Affairs Diabetes Trial (VADT) [99] have failed to replicate the cardioprotective results as reported in UKPDS [96], even with a well-controlled HbA_1c_ of <7% in T2DM. It is noteworthy, that the ACCORD study was prematurely terminated due to the unacceptably high mortality rate observed in T2DM individuals subjected to intensive glycaemic control [98]. Consistent with this finding, a recent meta-analysis revealed that intensive diabetic care enhance the risk of developing heart failure by 14% in diabetic patients when compared to those who received standard care [100]. Ultimately, an effective therapeutic regime for preventing the onset of DHD is clinically imperative, yet remains to be identified.

## Physical exercise as an intervention

Increased physical activity or active lifestyle has emerged as an effective therapeutic regimen to synergize the effects of pharmacotherapy in diabetic management and significantly reduce the risks of cardiovascular events [101–109], although the precise molecular mechanism(s) of action remain unclear. The American Diabetes Association (ADA) and the Diabetes Prevention Program (DPP) have advocated physical exercise as a non-pharmacological adjuvant to bolster the conventional management and prevention of DHD [110, 111]. A series of clinical and experimental studies has demonstrated that an appropriate volume and intensity of exercise can ameliorate myocardial dysfunction through the improvement of maximum oxygen consumption (VO_2max_), left ventricular ejection fraction (LVEF), LV diastolic and systolic volumes, ventilatory threshold, cardiac output and diastolic function (E/A ratio) [106, 108, 112–119].

## Exercise-mediated cardioprotection

**Fig. 2:**
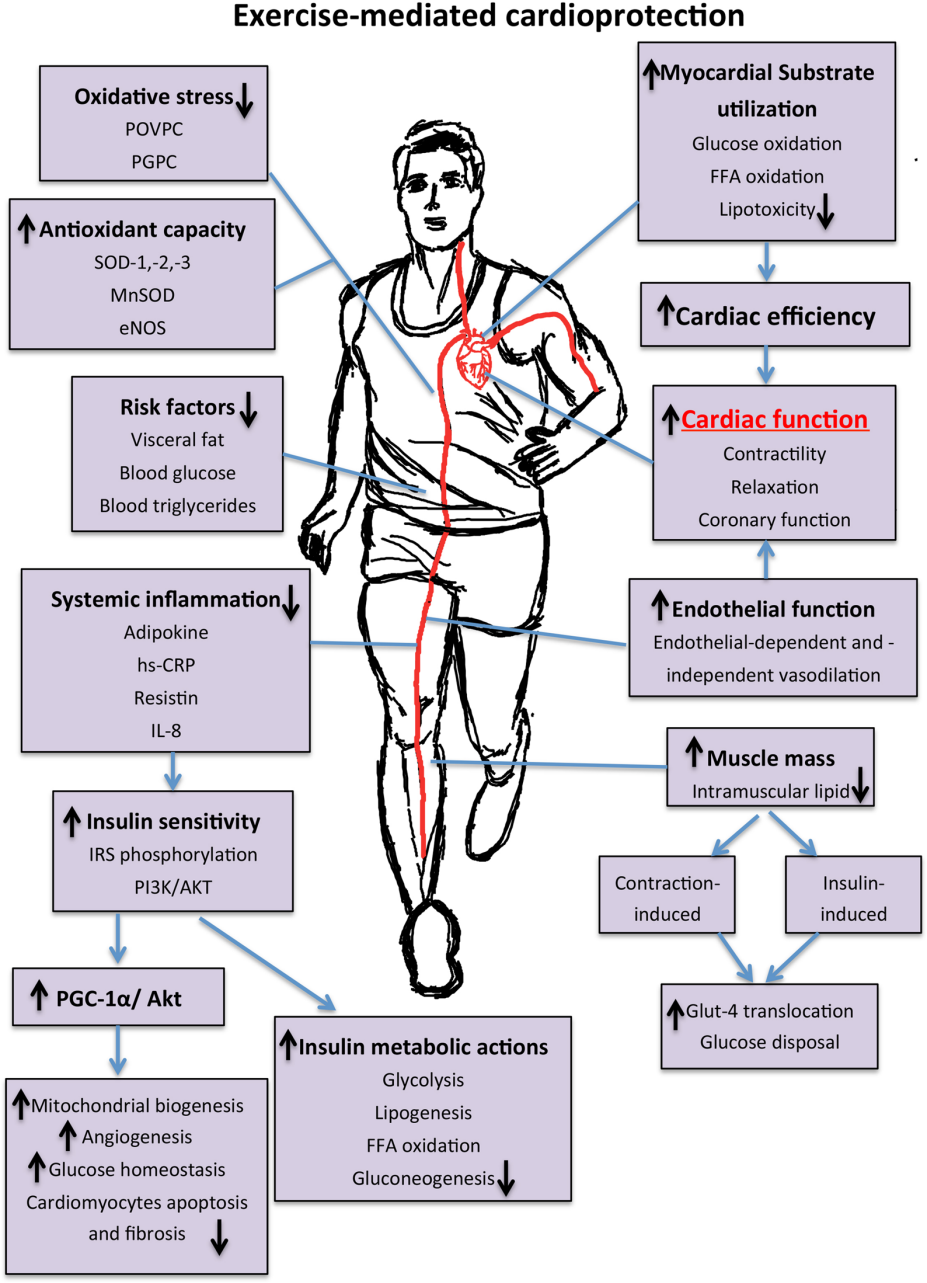
Exercise-induced cardioprotection through the modulation of (1) systemic risk factors, (2) endothelial and vascular functions and (3) cardiac performance directly

Exercise has been suggested to restore myocardial function through the improvement of VO_2max_, endothelial function, left ventricular systolic and diastolic function and blood pressure (Fig. 2) [106, 108, 109, 115, 120]. VO_2max_, a strong indicator for cardiorespiratory fitness and an independent predictor of cardiovascular mortality [121], was improved by 12–16% in obese postmenopausal women and obese individuals with T2DM in response to moderate-intensity exercise [122, 123]. Tjonna et al. [108] demonstrated that both moderate- and high-intensity exercise regimes were able to improve VO_2max_ of metabolic syndrome patients. Data on cardioprotective effects of low-intensity exercise is sparse. This could be due to the fact that low-intensity exercise may not meet the recommended minimum threshold of exercise intensity (e.g. >50% of VO_2max_) for improving cardiorespiratory endurance [124].

Cardiac tissue has an extremely high metabolic demand and, consequently, cardiac function is highly dependent on adequate coronary blood flow. Thus, coronary artery dysfunction directly impacts on optimal myocardial function. An eight-week moderate-intensity exercise regime in individuals with T2DM significantly improved endothelial function in the brachial artery as indicated by the improved flow-mediated dilation [125]. In another study, a 14-month moderate-intensity resistant training in adults with T2DM was able to improve the endothelial-dependent and -independent vasodilation in response to acetylcholine and sodium nitroprusside, respectively [126]. Of note, assessment of endothelial function in these studies was purely based on the brachial artery but not directly on the coronary endothelium, even though peripheral endothelial function has been suggested to correlate with function of the coronary endothelium [127, 128]. However, direct evidence of the beneficial effect of exercise on coronary artery function has been demonstrated in diabetic rodent models [113, 129, 130].

### Exercise mediated improvement of hyperglycaemia-induced cardiovascular dysfunction

As mentioned above, strict control of blood glucose with pharmacological intervention alone is not sufficient to reduce the risk of major cardiovascular events in people with T2DM. Indeed, intensive diabetic pharmacotherapy can even exacerbate cardiovascular events in diabetes patients [98, 100, 131]. Yet, exercise as an adjunct with anti-diabetic treatment (either with insulin or oral hypoglycaemic drugs) reduced the incidence of cardiovascular events [101–103], and improved VO_2max_ in T2DM patients [132]. Meta-analyses on the association between physical exercise with the risk of all-cause mortality and cardiovascular disease further demonstrated that increased physical activity was inversely correlated with cardiovascular risk and mortality in T2DM [133, 134]. Together, this evidence strongly advocates that active physical activity synergizes the effects of anti-hyperglycaemic drugs in the management of diabetic complications, in particular, cardiovascular dysfunction. The exact mechanism(s) that underpin this intriguing synergistic effect remain unclear.

Interestingly, the reported improvements of glycaemic control and myocardial function appear to be linked to a reduction in adiposity and an improvement in insulin sensitivity [122, 135–138]. Two recent randomized controlled trials reported that obese T2DM participants in weight loss programs showed improved glycaemic status and lowered triglycerides level, which was associated with a decreased risk of cardiovascular disease [139, 140]. Indeed, decreased visceral fat is associated with lower levels of adipokines that are crucial determinants in metabolic and vascular homeostasis (reviewed in [73]). Adipokines are known to negatively modulate SOCS3-mediated phosphorylation of IRS-1 protein. Therefore, exercise-mediated reduction in adipokines may restore activation of IRS-1 mediated PI3K-dependent signalling.

Moreover, improved insulin sensitivity can effectively elicit glucose disposal and reduce the amount of insulin required to maintain normal glucose levels [122, 135, 136]. Improved muscular strength [114, 141] and muscle density [122] following exercise can also facilitate glucose uptake independent of insulin action due to increased muscular contractions, which enhance translocation of GLUT-4 proteins to the skeletal muscle and sarcolemma (Fig. 2) [142]. Oguri and colleagues [143] demonstrated a significant increase of systemic glucose uptake in T2DM individuals and enhanced GLUT-4 membrane translocation in individuals after a single bout of moderate-intensity exercise Collectively, the evidence in the literature advocates that diabetic pharmacotherapy alone does not reduce the risks of cardiovascular events; rather the addition of routine physical exercise can synergise with the treatment through the improvement of insulin sensitivity and enhancement of glucose utilization.

### Exercise mediated improvement of insulin resistance-induced lipotoxicity

Using proton magnetic resonance spectroscopy (^1^H-MRS), Sai et al. [144] demonstrated a marked reduction in myocardial triglyceride content and a significant improvement in cardiac function in endurance athletes who were exercising for 5 days a week. In contrast, Schrauwen-Hinderling et al. [145] showed that combined endurance and strength training 3 times per week for 12 weeks improved insulin sensitivity, VO_2max_, LVEF and cardiac output in obese T2DM individuals without altering the cardiac lipid content. Although the design of the exercise training protocol would certainly impact on cardiac lipid content, it is also possible that improvement of cardiac function is independent of cardiac lipid content. Several other studies have also confirmed that a cardioprotective effect of exercise in diabetes is primarily through the improvement of myocardial substrate utilization by correcting the mismatch between myocardial FFA uptake and oxidative metabolism (Fig. 2) [112, 117, 146].

Hafstad et al. [112] reported an increase in myocardial glucose oxidation and a concomitant reduction in FFA oxidation in the hearts of *db/db* mice following a high-intensity treadmill exercise. This effect was accompanied by improved cardiac efficiency and cardiac mitochondrial respiratory capacity. Similarly, Broderick et al. [117] reported higher myocardial glucose oxidation and enhanced cardiac function after myocardial ischemia in diabetic rats subjected to high-intensity exercise. This might be attributable to an increase in sarcolemmal GLUT-4 proteins [147]. The increase in myocardial glucose uptake protects the heart by shifting away from over reliance on FFA oxidation.

Although it remains uncertain as to whether this experimental data can be translated into the clinical setting, due to limited clinical studies, there have been some studies that have provided important insight concerning the effect of exercise on cardiac function and glucose utilization in diabetic patients. For instance, endurance exercise in elderly men with impaired glucose tolerance improved total GLUT-4 protein expression, reduced intramuscular lipid content and increased FFA oxidation capacity in the vastus lateralis muscle [148]. With respect to myocardial substrate utilization, moderate-intensity exercise substantially increased myocardial lactate and glucose uptake and oxidation in young healthy subjects [149]. Sato et al. [150] suggested that exercise could reduce myocardial uptake rate of FFA and glutamate and enhance myocardial uptake of glucose, lactate and glutamate in ischemic heart disease patients.

### Exercise-mediated improvement of oxidative stress

There is an unambiguous relationship between oxidative stress and cardiac function (reviewed in [151, 152]). A considerable number of clinical studies have demonstrated the favourable effect of exercise on the systemic level of oxidative stress in diabetic individuals (Fig. 2) [153–159]. A recent randomized controlled trial on T2DM individuals who were subjected to 12-month of supervised aerobic, resistance and flexibility training demonstrated a reduction in plasma oxidative stress markers, 1-palmitoyl-2-(5-oxovaleroyl)-sn-glycero-3-phosphorylcholine (POVPC) and 1-palmitoyl-2-glutaroyl-sn-glycero-3-phosphorylcholine (PGPC) compared to those who received only standard medical care. Exercise also improved low-density lipoprotein (LDL) cholesterol, VO_2max_, insulin sensitivity and waist circumference [155]. Concomitantly, T2DM individuals who received 3 months of yoga therapy achieved a 20% reduction in malondialdehyde (MDA), a lipid peroxidation oxidative marker, compared to the non-exercise group. A decrease in oxidative stress was accompanied by enhanced glutathione and vitamin C and reduction in glycated haemoglobin (HbA_1c_) and fasting plasma glucose [156]. Again, this data strongly supports the notion that exercise has a synergistic effect as an adjuvant to further bolster the conventional therapeutic intervention in the management of diabetes.

Animal models of exercise have also consistently revealed significant improvements in cardiac antioxidant capacity and reduced oxidative stress (Fig. 2). Lee et al. [113] demonstrated an increase in the levels of antioxidants superoxide dismutase-1 and -2 (SOD-1 and SOD-2) and eNOS in the diabetic heart following a 10-week moderate-intensity aerobic exercise. This was associated with the improvement of coronary endothelial function in diabetic mice. Moreover, Moien-Afshari et al. [130] demonstrated a significant increase in mitochondrial SOD (MnSOD) and extracellular SOD (SOD-3) in the diabetic mice heart after an 8-week exercise regime.

The underlying mechanisms of how induction of antioxidants and restoration of redox status may benefit DHD are diverse. Some reports suggest that exercise is able to upregulate heat shock protein expressions to increase the antioxidant capacity in diabetes [160]. Exercise has also been shown to activate the antioxidant mediator, nuclear erythroid 2 p45-related factor 2 (Nrf2), a redox-sensitive transcription factor, to increase the expression of myocardial glutathione to buffer diabetes-induced oxidative stress. Depletion of Nrf2 abolished the expression of myocardial antioxidant genes such as catalase, glucose-6-phosphate dehydrogenase (*G6pd*), γ-glutamyl cysteine ligase-modulatory (*Gclm*), γ-glutamyl cysteine ligase-catalytic (*Gclc*), glutathione reductase (*Gsr*), and NAD(P)H-quinone oxydase-1(*Nqo1*) [161], thereby compromising the redox status in the myocardium. Furthermore, several studies have demonstrated an increase of ischemia-modified albumin (IMA) in T2DM individuals. IMA has been reported as an indicator of ischemic index, oxidative stress and peripheral arterial disease [162–164].

The long-term and regular incorporation of moderate-intensity exercise (e.g. walking) in T2DM individuals was able to prevent the increase in IMA and oxidative stress and hence reduce the risk of ischemia through induction antioxidants [154]. Once again, collectively, these data strongly suggested that a consistent and regular moderate-exercise regime is capable of buffering the hyperglycaemic- and insulin resistant-mediated oxidative stress in both the myocardium and systemic circulation, which could eventually prevent the development of diabetic cardiac dysfunction.

### Exercise-mediated protection against pro-inflammatory cytokines

It has been reported that exercise can reduce systemic inflammation in T2DM. An 18-year follow up study in middle-aged T2DM individuals who were highly involved in at least moderate-intensity physical exercise or greater reported a marked reduction in high-sensitivity C-reactive protein (hs-CRP), which was significantly associated with a reduction in total cardiovascular and coronary heart disease mortality [165]. Interestingly, exercise induced reduction in hs-CRP has been demonstrated to be related to an improved homeostatic model assessment-insulin resistance (HOMA-IR) index [123], an independent predictor of cardiovascular disease in T2DM [18] and also a measure of insulin resistance and β-cell function [166]. Obese T2DM individuals who underwent 16-week aerobic exercise training achieved a substantial reduction in resistin [123], a pro-inflammatory adipokine that is suggested to be associated with atherosclerosis and heart failure [167, 168]. This change was coupled with reductions in hs-CRP and IL-18 and improved VO_2max_.

As discussed above, proinflammatory cytokines suppress IRS-mediate insulin metabolic actions, which in turn are attributed to the cardiometabolic dysfunction. Therefore, improvement of the persistent low-grade systemic inflammation in the diabetic condition with exercise is believed to result from an improvement in insulin sensitivity by restoring the IRS-induced phosphorylation of PI3K-dependent pathway, thereby ameliorating the cardiovascular events.

### Exercise-mediated improvement of pro-survival signalling cascade

Restoration of altered pro-survival signalling cascade could play a major role in exercise-induced cardioprotection. As discussed above, impairment in insulin-mediated PI3K-dependent pathway alters the cell-signalling cascade. Therefore, restoration of this pathway can significantly improve insulin sensitivity and elicit insulin-associated metabolic effects by modulating glucose disposal, gluconeogenesis, lipogenesis and FFA oxidation (reviewed in [70, 73]). In animal models, endurance exercise has been shown to substantially improve insulin responsiveness through the phosphorylation of IRS-associated PI3K pathway, particularly IRS-1 and -2 proteins, Akt and its downstream substrate AS160 [169–173]. Other signalling pathways such as liver-kinase B1 (LKB1)-mediated phosphorylation of adenosine monophosphate-activated protein kinase (AMPK) pathway in skeletal muscle [174], adaptor protein phosphotyrosine interaction PH domain and leucine zipper containing 1 (APPL1)-mediated Akt pathway in liver [175] have also been reported to potentiate the metabolic actions of insulin.

Recently, Kjøbsted et al. [176] highlighted the physiological role of AMPK-Tre-2/BUB2/CDC16 domain family member 4 (TBC1D4) signalling axis in mediating the improvement of muscle insulin sensitivity after exercise. AMPK has also been suggested to play critical roles in regulating microvascular blood flow, glucose uptake and hence is considered to be a potential therapeutic insulin sensitizer [177].

Emerging evidence indicates the importance of peroxisome proliferator-activated receptor gamma coactivator 1-alpha (PGC-1α), a transcriptional coactivator, in exercise-mediated cardioprotection. PGC-1α is predominantly expressed in tissues with high oxidative capacity such as the heart, skeletal muscle, liver and brown adipose tissue, which has profound effects on mitochondrial biogenesis and energy metabolism [178–180]. Attenuated PGC-1α level leads to metabolic disorders. Adipose-specific PGC-1α deficiency in mice manifested the characteristics of T2DM, such as insulin resistance, impaired glucose tolerance and lipid metabolism, resulting in suppressed mitochondrial and thermogenic gene expressions in adipocytes [181]. Interestingly, the combined effects of enhanced PGC-1α expression and exercise training in mice improved glucose and insulin tolerance, suggesting a promising role for exercise-induced PGC-1α in treating metabolic disorders [182].

In addition to its effect on metabolism, Chinsomboon et al. [183] demonstrated the beneficial effects of exercise induced PGC-1α on angiogenesis. Using a genetic knockdown mouse model, they demonstrated that β-adrenergic stimulation during exercise is essential to induce activation of the PGC-1α/estrogen related receptor-α (ERRα) axis, which in turn upregulates vascular endothelial growth factor (VEGF) and platelet-derived growth factor (PDGF) expressions to markedly increase the capillary density in skeletal muscle [183]. Moreover, 15 weeks of moderate intensity treadmill exercise was also shown to attenuate diabetes-induced cardiac dysfunction and remodelling via PGC-1α and Akt activation in *db/db* mice, which possibly reduced myocardial apoptosis and fibrosis [173]. Taken together, induction of PGC-1α activation by exercise has various positive outcomes such as increasing insulin sensitivity, glucose transporters, as well as improving glucose homeostasis and fatty acid oxidation, [182, 184–187], all of which are important for the amelioration of the progression of DHD (Fig. 2).

Several clinical studies also support the notion that exercise training is able to enhance IRS-mediated PI3K-signalling in diabetic patients [188, 189]. Kirwan et al. [188] reported an enhancement of insulin-induced IRS-1-associated PI3K activation in vastus lateralis muscle of healthy aerobic exercise trained individuals compared to sedentary or untrained participants. The VO_2max_ was also 26% higher in trained individuals, reflecting a positive correlation between VO_2max_ and PI3K activation

Jorge et al. [190] compared the effects of aerobic, resistance and combined exercise on insulin signalling in T2DM individuals, and demonstrated 65 and 90% induction of IRS-1 expression in the skeletal muscles of resistance group and combined exercise group, respectively. Of importance, plasma glucose, blood pressure, systemic inflammatory cytokine and lipid profiles have improved in all groups of exercise [190]. Based on this evidence, restoration of IRS-mediated PI3K-dependent pathway could be one of the determining factors in mediating exercise-induced protection of the diabetic heart.

## Exercise-mediated differential expression of cardiovascular microRNAs

As stated above, metabolic dysregulation adversely triggers uncoupling of key cellular pathways from the very early stage of diabetes, which ultimately manifest as the functional and structural cardiac changes with the evolution of diabetes. These pathological changes appear to be closely associated with changes in the expression of microRNA (miR).

MiRs are small non-coding RNA molecules which are ~22 nucleotides long and regulate transcriptional and post-transcriptional gene expression (reviewed in [191]). A single miR can modulate complex pathological processes through their pleiotropic effects on multiple targets in disease development. The modulation of miRs in exercise-induced cardioprotection has received very little attention, yet it is an intriguing line of research that warrants urgent investigation. In the following section, we provide evidence to demonstrate that dynamic changes in miRs in response to exercise are able to induce significant cardioprotection. Table 2 summarizes the role of miRs in the development of cardiovascular disease and the following section will describe the role of each miR in detail.

**Table 2:** Summary of the known roles of miRs in the development of cardiovascular diseases [221]

MicroRNA	Expression in cardiovascular disease	Direct target(s)	Pathophysiological effect(s)	Reference(s)
MiR-1	Downregulated	JCN, Fbln2	Cardiac hypertrophy, remodelling, arrhythmias, cardiomyocyte apoptosis	[197, 207–209, 237]
MiR-133	Downregulated	CTGF, Bim, Bmf, Caspase-9	Cardiac remodelling, cardiomyocyte apoptosis	[192, 195–197]
MiR-499	Downregulated	Pdcd4, Pacs2, Dyrk2	Cardiomyocyte apoptosis	[210]
MiR-222	Downregulated	Hmbox-1, HIPK-1, HIPK-2, p27, p57	Cardiomyocyte apoptosis, cellular senescence, coronary artery disease, atherosclerosis	[211–214]
MiR-126	Downregulated	SPRED1, PIK3R2	Coronary artery disease, atherosclerosis, endothelial cell apoptosis	[215–217]

Junctin, JCN; Fibulin-2, Fbln2; Connective tissue growth factor, CTGF; B-cell lymphoma-2 like 11, Bim; B-cell lymphoma-2 modifying factor, Bmf; Programmed cell death 4, Pdcd4; Phosphofurin acidic cluster sorting protein 2, Pacs2; Dual specificity tyrosine phosphorylation regulated kinase 2, Dyrk2; Homeodomain interacting protein kinase, HIPK; Homeobox containing 1, Hmbox1; Sprout related EVH1 domain containing 1, SPRED; Phosphoinositide-3-kinase regulatory subunit 2, PIK3R2

### Exercise and muscle-specific miRs (miR-1, -133 and -499)

Several studies have proposed a potential role of miR-133 in the pathogenesis of heart disease [192–195], although its specific role in DHD still remains to be elucidated. Interestingly, the functional targets of miR-133, which include but are not limited to connective tissue growth factor (CTGF) [192, 195], B-cell lymphoma-2 like 11 (Bim), B-cell lymphoma-2 modifying factor (Bmf) [196] and Caspase-9 [197], have been extensively implicated in cardiac pathological remodelling and cell death. Hence, down-regulation of miR-133 has been associated with cardiac apoptosis, hypertrophy and myocardial matrix remodelling [192, 193, 197], all of which are known to induce adverse cardiac dysfunction in cardiovascular disease, possibly in DHD as well.

In a streptozotocin-induced diabetic cardiomyopathy mouse model, Chen et al. [195] reported an apparent link between a reduced myocardial expression of miR-133 and an augmented expression of fibrosis markers. Moreover, transgenic overexpression of miR-133 reversed diabetes-induced cardiac remodelling by attenuating these fibrotic markers. Concomitantly, in vitro induction of hypertrophy in cardiomyocytes by high glucose resulted in altered expression of miR-133 with enhanced expression of atrial natriuretic peptide (*Anp*) and brain natriuretic peptide (*Bnp*) mRNAs, indicators of pathological cardiac hypertrophy. The overexpression of miR-133 in neonatal rat cardiomyocytes attenuated the hypertrophic change via inhibition of serum and glucocorticoid-regulated kinase 1 (SGK1) and insulin-like growth factor 1 receptor (IGF1R) proteins [193].

**Fig. 3:**
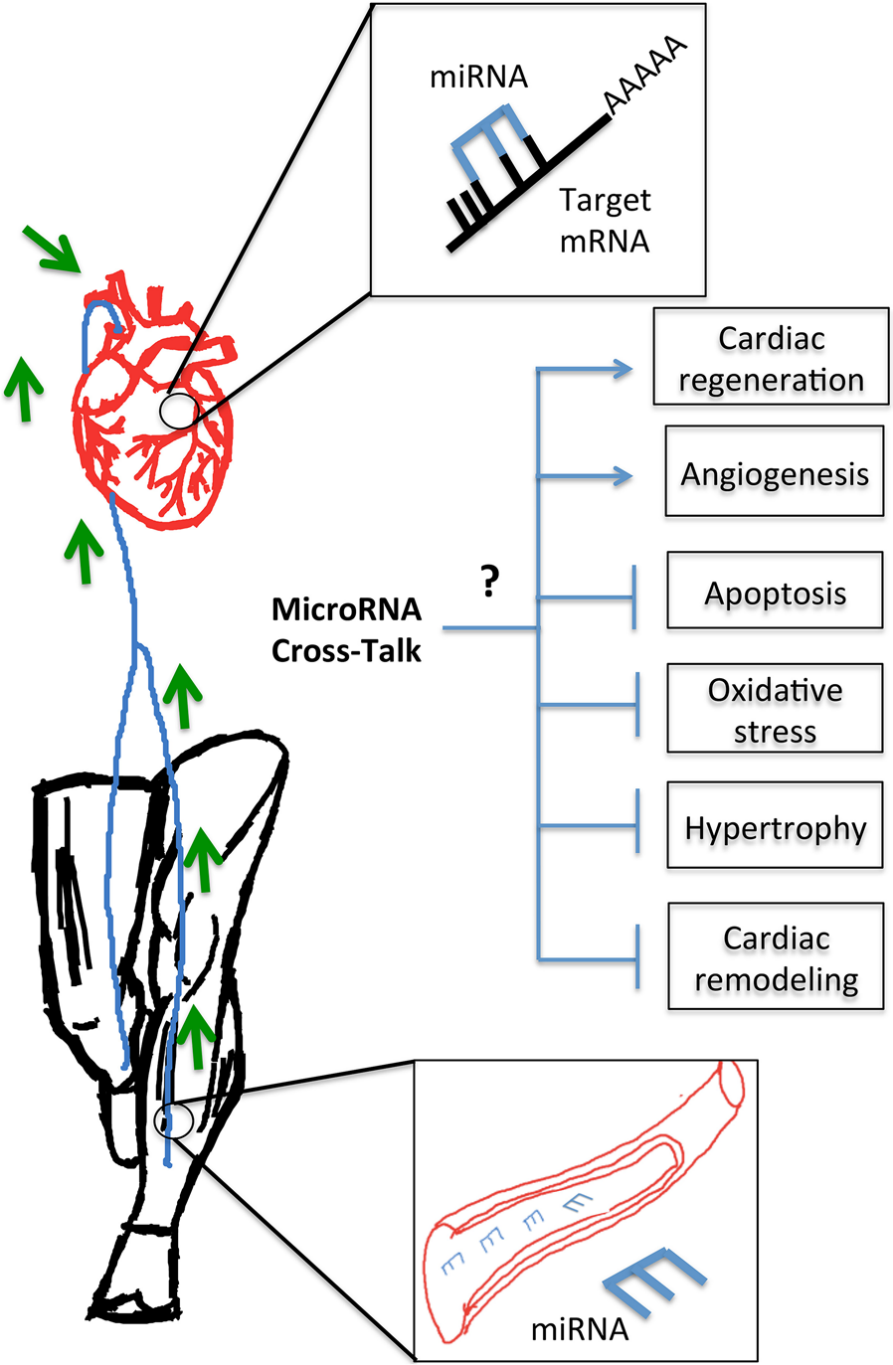
Proposed cross-talk effect between exercised skeletal muscle and cardiac muscles through microRNA communication

Although it is not clear whether exercise can normalize miR-133 expression in the myocardium, it is exciting to consider that exercise might be able to restore the expression of miR-133 by acting through a cross-talk effect. For example, both acute endurance and resistance exercise training in healthy male volunteers were able to enhance miR-133 expression in the vastus lateralis muscles [198, 199]. In addition, an increased level of miR-133 following marathon training appeared to be associated with improved VO_2max_ [200]. Moreover, endurance exercise elevated circulating levels of miR-133 in healthy individuals after either an acute bout of aerobic exercise, or endurance training [201, 202]. Similarly, subjecting T2DM mice to a 10-week swimming exercise regime increased the expression of miR-133 in cardiac tissue with improved contractile function and decreased matrix metallopeptidase-9 (MMP-9), an extracellular matrix regulator protein [203]. Since miR-133 is reported to be expressed and enriched in both cardiac and skeletal muscles (review in [191]), it is possible that miR-133 is secreted into the circulation from skeletal muscle after a bout of exercise, which then travels to the cardiomyocytes to suppress fibrotic markers and reduce cardiac hypertrophy (Fig. 3).

Frequently, skeletal muscle is referred as an endocrine organ due to its ability to release factors (myokines) that communicate with other distant organs or tissues through a ‘cross-talk’ effect to maintain homeostasis. For example, exercise has been shown to trigger the release of IL-6 from skeletal muscle and ultimately contribute to glucose metabolism [204, 205]. miRs can be released in a similar manner following exercise. Importantly our recent studies showed marked downregulation of muscle-specific miRs also known as myomiRs such as miR-1, -133 and -499 in the human diabetic heart [206]. Therefore, it is possible that exercise could activate the myomiRs in skeletal muscle which are released into the circulation (reviewed in [191]) which thereby restores the depleted level of myomiRs in the heart. While it is intriguing to speculate this cross-talk between skeletal muscle and the heart is one of the key molecular mechanisms underlying exercise induced cardioprotection (Fig. 3), available evidences support this hypothesis.

Aside from a potential ‘cross-talk’ effect of miRs, exercise could also have direct effects on cardiovascular miRs in diabetes. For instance, suppressed expression of miR-1, miR-133 and miR-499 in the STZ-induced diabetic rat heart was associated with augmented myocardial oxidative stress and cardiac dysfunction [207]. In particular, the direct target of miR-1: protein junctin, an integral protein of ryanodine receptor (RyR) in the endoplasmic/sarcoplasmic reticulum, was significantly elevated in the diabetic rat heart [207]. Previous studies on junctin overexpression transgenic mice demonstrated impaired calcium handling in cardiomyocytes, resulting in impaired cardiac relaxation, hypertrophy and arrhythmia [208, 209]. In addition, Wang et al. [210] has also confirmed that programmed cell death 4 (Pdcd4), phosphofurin acidic cluster sorting protein 2 (Pacs2) and dual specificity tyrosine phosphorylation regulated kinase 2 (Dyrk2) are direct targets of miR-499. Of which, Pdcd4 and Pacs2 are involved in hydrogen peroxide-induced apoptosis in H9c2 cells, a commonly used myoblast cell line. Hence, these lines of evidence strongly suggest that dysregulation of these miRs in diabetes can have profound adverse effects on the cardiac function.

Interestingly, treating diabetic rats with antioxidants for 4 weeks not only normalized all miRs, but also improved cardiac function and ultrastructure of the diabetic heart [207]. Since exercise is able to restore antioxidant defence and normalize oxidative stress, it is possible that exercise mediated normalization of miRs could have prevented the increase of their target proteins and oxidative stress eventually suppressing apoptosis and cardiac remodelling following exercise training. However, whether exercise protects the diabetic heart through the regulation of these miRs remains to be elucidated.

### Exercise and miR-222

MiR-222 is a highly conserved member of a miR cluster which is encoded on the X chromosome along with miR-221 and localized in the vascular wall of the vascular smooth muscle cells [211]. Liu et al. [211] identified the key role of miR-222 in vascular smooth muscle cell proliferation through its ability to suppress the inhibition of cyclin-dependent kinase. When the ECs and progenitor endothelial cells were challenged with highglucose and AGE (diabetes-like environment) in mice, this prevented the initiation of cell cycle and the migration of EC towards VEGF stimulation, which coincided with the downregulation of miR-221 and -222 [212]. Further, Jiang et al. [213] demonstrated marked downregulation of miR-222 along with miR-126 and miR-92a in patients with atherosclerosis, suggesting the importance of these miRs in the evolution of cardiovascular disease.

The mechanism of action of miR-222 is through inhibition of its target proteins: p27, homeodomain interacting protein kinase 1 (HIPK1), HIPK2 and homeobox containing 1 (Hmbox1) [214]. In addition, Togliatto et al. [212] validated p57 as another direct target protein of miR-222. p27 and p57 are inhibitors of cell cycle, therefore upregulation of these proteins indicate cellular senescence.

Liu et al. [214] are the first to demonstrate the role of miR-222 in exercise induced cardiac protection. They showed significant upregulation of miR-222 in cardiomyocytes of mice after swimming and voluntary running regimes. They further showed that inhibition of miR-222 resulted in increased cardiomyocyte apoptosis. Interestingly, overexpression of miR-222 in mice stimulated cardiomyocyte proliferation and growth [214], which was associated with an increased α/β myosin heavy chain (MHC) ratio as well as a reduction in ANP, BNP and α-skeletal actin mRNAs, indicating physiological cardiac adaptation. Importantly, cardiac-specific miR-222 transgenic mice were able to restore cardiac function, induce cardiomyocyte proliferation and reduce cardiac fibrosis after ischemia–reperfusion [214]. Taken together, exercise induced activation of miR-222 could be a powerful therapeutic strategy to replace the continuous loss of cardiomyocytes in individuals with diabetes.

### Exercise and miR-126

MiR-126, which is one of the most extensively studied miRs, is expressed in ECs and is a potent regulator of angiogenesis. The presence of miR-126 increases the pro-angiogenic VEGF protein through the inhibition of phosphoinositide-3-kinase regulatory subunit 2 (PIK3R2) and sprout related EVH1 domain containing 1 (SPRED1), inhibitors of VEGF. Suppression of the inhibitors by miR-126 leads to activation of PI3K and Raf-1 pathways, which ultimately promote VEGF activity [215, 216].

The knockdown of miR-126 expression in vivo induces leaky vessels, hemorrhage and loss of vascular integrity [215, 216]. Similarly, endothelial microparticles derived from high-glucose treated ECs showed limited endothelial cell migration and proliferation in vitro and reendothelialization in mice that had undergone carotid artery injury [217]. Moreover, a reduced expression of miR-126 has been linked to coronary artery disease, atherosclerosis and other vascular diseases [213, 217–220].

Recently we demonstrated that treatment of aortic rings from type-2 diabetic *db/db* mice and high-glucose treated Human Umbilical Vein Endothelial Cells (HUVECs) with miR-126 mimic markedly improved their impaired angiogenic potential by positively regulating VEGF protein. Increased cellular proliferation, cell migration and reduction of apoptosis were also observed as the positive outcomes of miR-126 treatment [221].

Exercise has enormous potential to be used as an approach to promote cardiac angiogenesis by stimulating the expression of miR-126 and VEGF protein. In support of this notion, a 5-day swimming regime for 10 weeks significantly increased myocardial capillary density in rats [222]. This beneficial effect was attributed to the elevation of VEGF/Raf-1/ERK and VEGF/P13 K/Akt pathways. Of note, exercise was able to repress SPRED1 and PIK3R2 proteins by upregulating miR-126 expression. Therefore, it is intriguing to postulate that exercise could be a cost-effective non-pharmacotherapy to potently upregulate angiogenic miR-126, which in turn may potentially improve coronary blood flow and function in DHD patients. More data in this context is needed to support our hypothesis.

## The potential for circulating miRs to determine exercise induced cardioprotection

Alterations in the expression of cardiac-specific miR’s in the early stage of diabetes may implicate the development of DHD. Rawal et al., demonstrated dysregulated expression of cardiac-specific miRs in diabetic individuals with normal EF, and further suggested that this early dysregulation together with some other clinically detectable changes may act as a catalyst for the clinical manifestations of DHD [206]. In line with this, several clinical and experimental studies have implicated the importance of miR dysregulation in cardiovascular diseases [194, 223–228]. Interestingly, miRs are released into the circulation either packed as microvesicles or bound to lipoprotein molecules where they remain stable [229, 230]. This property of miRs gives them potential as diagnostic and prognostic biomarkers for cardiovascular diseases to identify the disease in the early stage (reviewed in [191]).

A 15-year follow-up study by Zampetaki et al. [218] revealed that the expression of miRs-15a, -29b, -126 and -223 were adversely altered from the early stages of diabetes well before the development of clinical manifestations. Moreover, other studies also demonstrated the dynamic changes of miR in response to therapeutic treatment, thereby suggesting the potential role of miR as a prognostic marker for the disease [231–233].

As described above, one of the major limitations for the sustaining exercise regime is the inability to demonstrate immediate benefit. However, it is possible to demonstrate the immediate benefit of exercise through serial measurement of changes in miRs, which we consider will have a strong impact on whether individuals can sustain exercise in the long term. To support this notion, a recent study reported that circulating miR-1, -133 and -206 remained elevated for up to 24 h after running a marathon. The authors demonstrated a positive correlation between the miRs and VO_2max_ and individual anaerobic lactate threshold (VIAS). Importantly, none of these miRs were associated with cardiac injury markers such as cardiac troponin T, troponin I and pro-BNP [200].

## Therapeutic potential of microRNA in DHD

Since the discovery of miRs, some two decades ago, a plethora of studies have investigated the adverse and beneficial effects of miRs in different types of diseases using diverse in vivo and in vitro models. Ultimately, select miR’s have been targeted by pharmaceutical companies as potential pathways for treating diseases. For instance, miR-15, -208 and -92a are currently undergoing intensive pre-clinical trials for preventing the deleterious effects associated with myocardial infarction, hypertrophic cardiomyopathy and heart failure as well as peripheral artery disease [234]. Of note, the miR-29b mimic (MRG201-30-001), which attenuates pathological fibrosis, has now entered clinical trial phase I [234], while the miR-122 antagonist (SPC3649), is now in clinical trial phase II for the treatment of hepatitis C [235].

Although miR’s appear as a new and exciting therapeutic strategy, only few miRs are currently undergoing clinical trials. One important reason for the cautionary approach in trialling miRs/anti-miRs in patients is that some miR’s have pleiotropic effects so that inhibition or overexpression of one particular miR could have other off-target effects on non-specific organs. Therefore, it is critical to generate toxicology and safety data for miR’s to compare with the current standard treatments in order to build a solid safety profile of any particular miR. Interestingly, the miR’s that are currently in clinical trials are organ- or tissue-specific.

To date, there is no single miR candidate that has entered clinical trials for the treatment of DHD. As described above miR-1, -133, -222 and -126 are known to possess potent cardioprotection properties in animal models of diabetes. However, efficacy data, pharmacokinetics and toxicology profiles of these miRs are needed to justify clinical trials assessing these miRs in DHD. Ultimately, the research and clinical communities are enthusiastic to unravel the roles of miRs as powerful and promising interventions.

## Future directions and concluding remarks

Despite significant advances in medical research and the long clinical history of anti-diabetic treatments, the incidences of diabetes-related cardiovascular complications are increasing exponentially. Yet, with no specific and effective pharmacological treatment, DHD is one of the major contributors to increased mortality rate in the current diabetic population. Results from clinical trials show that the strict control of glycemic status alone is ineffective at preventing diabetes-induced cardiac dysfunction and, in contrast, can potentially aggravate dysfunction. Surprisingly, a large number of controlled clinical trials demonstrated synergistic effects of exercise in conjunction with pharmacological treatments for managing glycaemic status in diabetes, and attenuated risks of developing cardiac dysfunction.

Although the efficacy of anti-diabetic drug regimes might be limited in ability to prevent DHD, exercise is emerging as having addictive effects for improving hyperglycaemia and insulin sensitivity by normalizing myocardial oxidative stress, lipotoxicity and systemic inflammation in diabetes. On the basis of strong evidence from molecular studies, others and we advocate exercise for ameliorating the progression of diabetes-associated cardiac dysfunction. However, as the long-term sustainability of a ‘high-intensity’ exercise regime has not been reported, it is reasonable to suggest that the ability of a diabetic (and often obese) population to sustain exceptionally high intensity levels of exercise might be a concern. For instance, supervised short-term high-intensity exercise has been suggested to be superior to moderate-intensity exercise in improving myocardial functions [108, 120]. However, given that the diabetic population frequently have poor exercise capacity or low VO_2max_, it may not be practical or realistic for diabetics to incorporate high-intensity exercise as lifestyle change for the prevention of DHD. This is further supported by the fact that adherence to exercise is inversely proportional to exercise intensity [236].

Yet, the emergence of miR as new and exciting biomarkers could aid clinicians in identifying those diabetic patients who are at higher risk, or predisposed to DHD at an early stage of disease. In doing so, it is hoped that a relatively modest, and sustainable, level of exercise could be prescribed to patients as an effective prophylactic strategy against DHD. Moreover, it is possible to use miRs as a marker to demonstrate the immediate benefit of exercise on heart, which is currently impossible by other means.

Based on our current understanding of the benefits of exercise on cardiac function in diabetes, there is a sense of urgency that future studies focus on the optimal intensity of exercise in diabetic patients, in the very early stages of the disease, that is both sustainable long-term as a lifestyle adaptation, but also effectively protects against the onset of DHD because, ultimately, DHD is a chronic condition that will require life-long management for ensuring a successful outcome.

## Abbreviations

T2DM: type-2 diabetes mellitus
FHS: Framingham Heart Study
CDH: coronary heart disease
DMD: diabetic heart disease
NIH: National Institute of Health
PKC: protein kinase-C
AGEs: advanced glycation end products
NADPH: nicotinamide adenine dinucleotide phosphate
GSH: reduced glutathione
DAG: diacylglycerol
G3P: glycerol-3-phosphate
NF-κB: nuclear factor kappa-light-chain-enhancer of activated B cell
eNOS: endothelial nitric oxide synthase
ROS: reactive oxygen species
RAGE: AGE receptor
ECs: endothelial cells
GlcN-6-P: glucosamine-6-phosphate
GFAT: glutamine:fructose-6-phosphate amidotransferase
UDP: uridine diphosphate
O-GlcNAc: O-linked *N*-acetylglucosamine transferase
IRS-1: insulin receptor substrate-1
PI3K: phosphoinositide-3 kinase
PDK1: phosphoinositide-dependent kinase 1
Akt: protein kinase B
aPKC: atypical protein kinase C
GLUT: glucose transporter
MAPK: mitogen-activated protein kinase
JNK: C-Jun-N-terminal kinase
ERK: extracellular signal-regulated kinase
FFA: free fatty acid
TNF- α: tumour necrosis factor-alpha
IL: interleukin
NFκB: nuclear factor kappa B
IKKβ: I-kappaB kinase beta
SOCS: suppressor of cytokine-signalling
UKPDS: United Kingdom Prospective Diabetes Study
ADVANCE: action in diabetes and vascular disease
ACCORD: action to control cardiovascular risk in diabetes
VADT: Veterans affairs diabetes trial
ADA: American Diabetes Association
DPP: diabetes prevention program
VO_2max_: maximum oxygen consumption
LVEF: left ventricular ejection fraction
miR: microRNA
^1^H-MRS: proton magnetic resonance spectroscopy
POVPC: 1-palmitoyl-2-(-5-oxovaleroyl)-sn-glycero-3-phosphorylcholine
PGPC: 1-palmitoyl-2-glutaroyl-sn-glycero-3-phosphorylcholine
LDL: low-density lipoprotein
MDA: malondialdehyde
HbA_1_: glycated haemoglobin
SOD: superoxide dismutase
MnSOD: mitochondrial SOD
Nrf2: nuclear erythroid 2 p45-related factor 2
*G6pd*: glucose-6-phosphatedehydrogenase
*Nqo1*: NAD(P)H-quinone oxydase-1
*Gsr*: glutathione reductase
*Gclc*: ϒ-glutamyl cysteine ligase-catalytic
*Gclm*: ϒ-glytamyl cysteine ligase-modulatory
IMA: ischemia-modified albumin
hs-CRP: high-sensitive C-reactive protein
HOMA-IR: homeostatic model assessment-insulin resistance
LKB1: liver-kinase B1
AMPK: adenosine monophosphate-activated protein kinase
APPL1: adaptor protein phosphotyrosine interaction PH domain and leucine zipper containing 1
PGC1-α: peroxisome proliferator-activated receptor gamma coactivator 1-alpha
ERRα: estrogen related receptor- α
VEGF: vascular growth factor
PDGF: platelet-derived growth factor
CTGF: connective tissue growth factor
Bim: B cell lymphoma-2 like 11
Bmf: B-cell lymphoma-2 modifying factor
*Anp*: atrial natriuretic peptide
*Bnp*: brain natriuretic peptide
SGK1: serum and glycocorticoid-regulated kinase 1
IGF1R: insulin-like growth factor 1 receptor
MMP-9: matrix metallopeptidase-9
JCN: junctin
RyR: ryanodine receptor
Pdcd4: programmed cell death 4
Pacs2: phosphofurin acidic cluster sorting protein 2
Dyrk2: dual specificity tyrosine phosphorylation regulated kinase 2
MHC: myosin heavy chain
HIPK: homeodomain interacting protein kinase
Hmbox1: homeobox containing 1
PIK3R2: phosphoinositide-3-kinase regulatory subunit 2
SPRED1: sprout related EVH1 domain containing 1
HUVECs: human Umbilical Vein Endothelial Cells
VIAS: individual anaerobic lactate threshold
